# Supplementary material for: Evaluation of gene expression of PLEKHS1, AADAC, and CDKN3 as novel genomic markers in gastric carcinoma
Source: PLoS One. 2022 Apr 21;17(4):e0265184. doi: 10.1371/journal.pone.0265184 (PMC9022821; doi:10.1371/journal.pone.0265184)
Supplement: Attachment — Submitted filename: Response to Reviewers.docx [file pone.0265184.s002.docx]

**Reviewer #1**

1. The rationales for choosing PLEKHS1, AADAC and CDKN3 as targets are missing in introduction or discussion.

Response:

The rationales for choosing PLEKHS1, AADAC and CDKN3 as targets are included in the following paragraph in the introduction “A bioinformatics analysis study on GC found PLEKHS1 as a protective prognostic gene and AADAC as a risky prognostic gene while CDKN3 was found to be tightly correlated with the pathogenesis of GC [13], so the present study intended to experimentally evaluate these findings.”

2. By emphasizing Egyptian patients in the study, are there any difference for the expression profile of PLEKHS1, AADAC and CDKN3 between Egyptian patients and patient from other area?

Response:

We do not know if there are any difference for the expression profile of PLEKHS1, AADAC and CDKN3 between Egyptian patients and patient from other area so, we delete these words “in Egyptian patients “from the title.

3. The association between PLEKHS1, AADAC, and CDKN3 are missing. Moreover, the molecular mechanism for the impacts of PLEKHS1, AADAC, and CDKN3 in tumor is not showed**.**

Response:

A high significant negative correlation between AADAC, and CDKN3 gene expression (r= -.760, p<0.001) was found. This paragraph was added to the abstract, results, and discussion. The molecular mechanism for the impacts of PLEKHS1, AADAC, and CDKN3 in tumor needs more evaluating further future studies as we mentioned at the end of the abstract and conclusion.

**Reviewer #3**

1. The authors need to provide a clear definition of their sample set like inclusion and exclusion criteria. For instance, they need to provide information on the source of their normal samples. Paired normal (Sourced from adjacent normal tissues away from the tumor) or normal gastric tissue. It would be ideal if they could compare with both kinds of normal tissue. Histological subtype of the samples assessed. The overall distribution of samples across the various stages is highly limited. For instance, only 2 samples are used in stage IV. In table 1 it is not clear what the authors mean the by the term “Outcome”. The row on Metastasis needs to describe which organ. Visceral organ metastasis is considered stage IV disease.

Response:

We mentioned that the source of the normal samples were adjacent normal tissues away from the tumor.

We mean by outcome in table 1; tissues have tumor (positive) or not (negative) and we delete this item from the table as if it could not be understood.

only 2 samples are used in stage IV because we were eager to have fresh frozen samples but at this time, only 2 samples in stage IV were available.

Metastasis were found as 14 samples obtained from patients with bone metastasis, 4 samples obtained from patients with bone and brain metastasis, and 2 samples obtained from patients with bone and liver metastasis

2. The tissue source is from archival FFPE sections, though routinely used for analysis there are some drawbacks regarding the quality of RNA sourced from this source. Preferably including fresh frozen samples in the study will help in overcome questions regarding the quality of the assays performed.

Response:

The samples already were fresh frozen samples just stored in the pathology department repositories at the time of collecting the samples for the present work.

3. Additional protein expression studies using Immunohistochemistry and archival FFPE sections for the markers will considerably increase the impact of the findings by increasing the sample numbers.

Response: These additional protein expression studies need more cost, and we have no any funding source except our own work, so we add that our findings need more evaluating further future studies to overcome all limitations in our study.

4. In the results section the authors mention fold change values “[(0.89±0.124), and (0.80±0.09)] compared to the corresponding values in the control group [(0.41±0.04), and (0.43±0.126) respectively] “what the common calibrator was used to determine the fold changes in tumor and control groups?

Response: The common calibrator was used to determine the fold changes in tumor and control groups expressed by (M±SD), these words are added to the result section.

5. It is not clear why the authors mention “Demographic Data in the studied groups:” considering the fact that the control samples were sourced from the same gastric tumor tissue (paired normal) else the authors need to specifically mention this.

Response: We mean demographic data in the studied subjects not groups. We delete this paragraph to avoid misunderstanding from readers.

6. While mentioning data on “Relations between the tumor progression and the studied parameters” it is necessary to give background information on how many of the patients progressed during treatment and how many did not do so.

Response: All the patients in our study progressed during treatment. We add these words to the subject and methods section.

7. Overall, considering that the present study is a validation analysis a larger sample set is required to assess the markers along with clinicopathological features such as H. Pylori status, subtypes of gastric cancer.

Response:

You are right! this is a one of our study limitations, but these additional data were not available for all patients, so we could not do this assessment.

**Reviewer #4**

1. Initially, English should be revised, and the tittle "Figure Ligands" must be corrected to "Figure legends".

Response: It is done

2. These should also be improved and include more descriptive data on the figures to which they refer.

Response: It is done

3. The style of the References must be corrected and adapted to the standard recommended by the Journal.

Response: It is done

4. With respect to the Subjects and Methods section, the number of specimens analyzed in some groups is low (e.g., n=2 for G1 tumor grade and tumor stage IV)

Response:

only 2 samples are used in stage IV or in grade 1 because we were eager to have fresh frozen samples but during the period of our samples collection, only theses samples were available.

5. Unfortunately, only 1 housekeeping gene was used (ideally authors should work with 2 housekeeping genes) and another technique that allows the assessment of proteins that are encoded by the analyzed genes (such as IHC) should be added in order to strengthen the results.

Response:

These additional protein expression studies need more cost, and we have no any funding source except our own work, so we add that our findings need more evaluating further future studies to overcome all limitations in our study.
