# Supplementary material for: Evaluation of gene expression of PLEKHS1, AADAC, and CDKN3 as novel genomic markers in gastric carcinoma
Source: PLoS One. 2022 Apr 21;17(4):e0265184. doi: 10.1371/journal.pone.0265184 (PMC9022821; doi:10.1371/journal.pone.0265184)
Supplement: Attachment — Submitted filename: Response to reviewer 4.docx [file pone.0265184.s003.docx]

**Response to reviewers**

**Reviewer #4**

1. Initially, English should be revised, and the tittle "Figure Ligands" must be corrected to "Figure legends".

**Response**: It is done

2. These should also be improved and include more descriptive data on the figures to which they refer.

**Response**: It is done

3. The style of the References must be corrected and adapted to the standard recommended by the Journal.

**Response**: It is done

4. With respect to the Subjects and Methods section, the number of specimens analyzed in some groups is low (e.g., n=2 for G1 tumor grade and tumor stage IV)

**Response:**

GC has low incidence and prevalence in Egyptians representing 1.6% of the total cancers [1], so we could not find more fresh frozen samples during the period of our samples collection. We were eager to work as rapid as possible to ensure the stability of RNA in the collected samples only 2 samples were used in stage IV or in grade 1 because, only theses samples were available in the fresh frozen samples during the period of our samples collection.

5. Unfortunately, only 1 housekeeping gene was used (ideally authors should work with 2 housekeeping genes) and another technique that allows the assessment of proteins that are encoded by the analyzed genes (such as IHC) should be added in order to strengthen the results.

**Response:**

Using of one housekeeping gene is the routine method at our molecular biology unit of Kasr Alaini Medicine College in Egypt. This method is accepted in many published articles introduced by researchers to international journals and PLoS One journal [2].

These additional protein expression studies need more cost, and we have no any funding source except our own work, so our study evaluates the gene expression ant it is obvious in the title of the article mentioning that that our findings need more confirming further future studies to overcome all limitations in our study. It is very difficult to do additional work on the same samples.

**References**

1.Abdelwahab Hashem TA, El-Fotouh MA, Ehab A, El Rebey HS, Satar MA, Attallah HS. Her-2 neu status in gastric carcinoma in Egyptian patients: The epidemiology and the response to chemotherapy. Menoufia Med J 2016; 29: 449-53.

2. Shan L, Lian F, Guo L, Qiu T, Ling Y, Ying J, Lin D. Detection of ROS1 gene rearrangement in lung adenocarcinoma: comparison of IHC, FISH and real-time RT-PCR. PLoS One. 2015 Mar 5;10(3):e0120422. doi: 10.1371/journal.pone.0120422. PMID: 25742289; PMCID: PMC4351102.
