# Supplementary material for: Evaluation of gene expression of PLEKHS1, AADAC, and CDKN3 as novel genomic markers in gastric carcinoma
Source: PLoS One. 2022 Apr 21;17(4):e0265184. doi: 10.1371/journal.pone.0265184 (PMC9022821; doi:10.1371/journal.pone.0265184)
Supplement: Attachment — Submitted filename: Response to reviewer 4.docx [file pone.0265184.s004.docx]

**Response to reviewers**

**Reviewer #4**

1. The style of the References must be corrected and adapted to the standard recommended by the Journal.

**Response**: I reformatted, revised all references through EndNote X9 and corrected all mistakes in the references.
